# Midpregnancy Placental Growth Factor Screening and Early Preterm Birth

**DOI:** 10.1001/jamanetworkopen.2024.44454

**Published:** 2024-11-14

**Authors:** Rachel A. Gladstone, Sumaiya Ahmed, Ella Huszti, Kelsey McLaughlin, John W. Snelgrove, Jennifer Taher, Sebastian R. Hobson, Rory C. Windrim, Kellie E. Murphy, John C. Kingdom

**Affiliations:** 1 Departments of Obstetrics & Gynecology, Maternal-Fetal Medicine Division, University of Toronto, Toronto, Ontario, Canada; 2 Department of Obstetrics & Gynecology, University of Toronto, Toronto, Ontario, Canada; 3 Institute of Health Policy, Management, and Evaluation, University of Toronto, Ontario, Canada; 4 Institute of Medical Science, University of Toronto, Toronto, Ontario, Canada; 5 Pathology and Laboratory Medicine at Mount Sinai Hospital, University of Toronto, Toronto, Ontario, Canada

## Abstract

**Importance:**

Early preterm birth (ie, at less than 34 weeks’ gestation) confers a high risk for adverse health outcomes, yet no universal screening strategy exists, preventing targeted delivery of effective interventions.

**Objective:**

To evaluate the ability of midpregnancy placental growth factor (PlGF) screening to identify pregnancies at highest risk for early preterm birth.

**Design, Setting, and Participants:**

This prospective cohort study was conducted at an urban, tertiary care center from 2020 to 2023. Participants were unselected, pregnant people with singleton pregnancies, receiving universal-access prenatal care from obstetricians, family physicians, or midwives, who underwent a PlGF test at the time of routine gestational diabetes screening, typically at 24 to 28 weeks’ gestation. Data were analyzed from January to May 2024.

**Exposure:**

PlGF level less than 100 pg/mL at the time of gestational diabetes screen.

**Main Outcomes and Measures:**

The primary outcome was all early preterm birth, defined as less than 34 weeks’ gestation. Secondary outcomes included iatrogenic preterm birth, spontaneous preterm birth, preeclampsia, stillbirth, and small-for-gestational-age birth weight.

**Results:**

Among 9037 unique pregnant individuals, 156 (1.7%) experienced early preterm birth (52 spontaneous births; 104 iatrogenic births). The area under the curve (AUC) for PlGF and early preterm birth was 0.80 (95% CI, 0.75-0.85). Low PlGF level was associated with early preterm birth (positive likelihood ratio [LR], 79.400 [95% CI, 53.434-115.137]; negative LR, 0.606 [95% CI, 0.494-0.742]; specificity, 99.5% [95% CI, 99.3%-99.6%]; negative predictive value, 98.9% [95% CI, 98.8%-99.1%]). Time to birth from PlGF test was significantly reduced among patients with a PlGF level less than 100 pg/mL, among whom more than 50% delivered within 50 days of testing. Individuals with a low PlGF level made up more than 30% of subsequent stillbirths (aRR, 36.78 [95% CI, 18.63-72.60]) and more than half of patients requiring iatrogenic early preterm birth (aRR, 92.11 [95% CI, 64.83-130.87]). The AUC for iatrogenic early preterm birth was 0.90 (95% CI, 0.85-0.94).

**Conclusions and Relevance:**

These findings suggest that low PlGF level (<100 pg/mL), identified at the time of routine gestational diabetes screening, may be a powerful clinical tool to identify pregnant people at risk of early preterm birth, especially in iatrogenic births. Strategic redirection of tertiary health care resources to this high-risk group could improve maternal and perinatal outcomes.

## Key Points

**Question:**

Is placental growth factor (PlGF) testing at the time of gestational diabetes screening among unselected, singleton pregnancies associated with higher risk for early preterm birth (ie, <34 weeks)?

**Findings:**

In this cohort study of 9037 independent singleton pregnancies, screening PlGF level less than 100 pg/mL was associated with 79.4-fold increased likelihood of early preterm birth. Low PlGF level was present in more than half of iatrogenic early preterm births and one-third of all antepartum stillbirths.

**Meaning:**

These findings suggest that unimodal, midpregnancy PlGF testing may serve as a candidate screening tool for identifying pregnant people at highest risk for early preterm birth.

## Teaser

This cohort study evaluates the association of placental growth factor (PlGF) level measured at midpregnancy to identify pregnancies at the highest risk for early preterm birth.

## Introduction

Preterm birth, defined as prior to 37 weeks of gestation, affects approximately 9% of singleton births and is the leading cause of death before age 5 years.^1,2^ One-quarter of these births occur before 34 weeks, constituting early preterm birth, which is associated with the greatest risk of adverse infant and childhood outcomes, at tremendous societal cost.^1,3,4^ While the burden of preterm birth is disproportionately heavy in low- and middle-resource countries, it remains a major public health concern in higher-resource nations, where preterm birth and infant mortality rates remain high.^5,6,7,8^

Preterm birth occurs either spontaneously, including with preterm premature rupture of the membranes, or as a result of medical intervention, by induction of labor or planned Cesarean birth, typically to either reverse potentially life-threatening severe preeclampsia or to prevent impending stillbirth from fetal growth restriction (FGR).^9^ More than one-half of all preterm births are iatrogenic.^10^ In their more severe forms associated with iatrogenic preterm birth, both preeclampsia and FGR are strongly associated with underlying chronic placental vascular pathology, termed *maternal vascular malperfusion*, which greatly impairs the ability of the placenta to release placental growth factor (PlGF) into maternal circulation.^9,11^ In healthy pregnancy, PlGF increases progressively, to a peak at 28 to 30 weeks, augmenting the vasodilatory, proangiogenic actions of vascular endothelial growth factor and supporting physiologic systemic vascular relaxation.^12,13^ PlGF is suppressed in most pregnant individuals requiring iatrogenic preterm birth for severe preeclampsia or FGR at the time of clinical diagnosis.^12,13,14,15^

Effective identification of early preterm birth risk could facilitate implementation of established medical interventions to optimize maternal and perinatal health. These include transfer for specialized perinatal care and timely maternal administration of medications, such as corticosteroids, magnesium sulfate, or antihypertensives, where relevant. An estimated 75% of preterm birth–related neonatal and childhood deaths are preventable with available, evidence-based interventions.^16^ Lack of recognition of populations at risk of early preterm birth may in part explain why severe maternal and perinatal adverse outcomes continue to occur at unacceptable levels, while maternal mortality rates continue to rise.^17,18^

Measurement of PlGF level in maternal blood is an established component of first trimester, multimodal screening for early preterm birth associated with preeclampsia in the UK.^19^ A comparable multicountry approach in the second trimester achieved similar results,^20^ yet neither strategy has been widely adopted into clinical practice. Currently, no unimodal screening test exists for early preterm birth in the general population. Therefore, we evaluated maternal circulating PlGF as a midpregnancy screening test for all preterm births at less than 34 weeks’ gestation in a large, prospective cohort of unselected, singleton pregnancies.

## Methods

### Study Design

We conducted a single-center, prospective cohort study at Mount Sinai Hospital, a tertiary care center in Toronto, Canada, between April 2020 and April 2023. The research ethics board (REB) of Mount Sinai Hospital approved this study. As enrollment was conducted on an opt-out basis, the REB determined further informed consent was not required. The findings are reported following the Strengthening the Reporting of Observational Studies in Epidemiology (STROBE) reporting guideline.

### Participants

Pregnant people aged 18 years or older with viable, singleton pregnancies under the care of obstetricians, family physicians, or midwives at Mount Sinai Hospital were included. Recruitment from this diverse, predominantly low-risk pool of patients proceeded using an information leaflet provided at the time of screening for gestational diabetes, with opt-out enrollment, per REB approval. All participants received uniform, publicly funded care. Individuals with preexisting signs of placental dysfunction (eg, preeclampsia, FGR), multiple gestations, pregestational diabetes, and kidney disease and those who ultimately gave birth outside of Mount Sinai Hospital were excluded.

### Procedures

Eligible participants underwent PlGF blood testing at the time of gestational diabetes screening, generally at 24 to 28 weeks’ gestation, although participants tested within 2 weeks of this range were also included (22-30 weeks). Daily PlGF testing occurred in the hospital central laboratories (Elecsys, Roche Diagnostics). Results were posted to the hospital electronic medical record (EMR) within 2 hours of sampling. In the interest of patient safety during the COVID-19 pandemic, REB approval was conditional on values less than 100 pg/mL being flagged for review, given previous experience with PlGF testing for diagnostic purposes in high-risk obstetric populations in both the UK^21^ and Canada.^22^ This typically prompted fetal ultrasonographic surveillance and enhanced monitoring for maternal hypertension. Following birth, maternal demographics, laboratory values, vital signs, and delivery information were collected from the EMR.

In the rare event that an individual gave birth more than once within the study period, only the first eligible pregnancy was included in the analysis, to maintain independence of observations. Where multiple PlGF tests were performed between 24 and 28 weeks, the earliest result was analyzed. If no PlGF result was available between 24 and 28 weeks, the next closest PlGF test within 2 weeks was included in the analysis. Gestational age–adjusted PlGF percentiles were calculated in keeping with our group’s previous work.^13^

Maternal race data were abstracted from self-reported categorical data fields within the EMR, including Asian or South Asian, Black, White, other, and unknown. The race category other was selected by participants who did not self-identify into any specific category. Reporting of race was not a mandated EMR field and thus was coded as unknown if not completed. Race data were collected because non-White racial categories are associated with higher rates of preterm birth in comparison with individuals identifying as White.

The primary outcome was all early preterm birth, defined as less than 34 weeks’ gestation, of a live or stillborn fetus. The secondary outcomes were gestational age at birth, all preterm birth at less than 37 weeks’ gestation, spontaneous preterm birth, iatrogenic preterm birth, stillbirth, preeclampsia, severe preeclampsia, HELLP (hemolysis, elevated liver-enzyme levels, and low platelet count) syndrome, small-for-gestational-age (SGA) birth weight (<10th percentile and <3rd percentile), and mode of birth.

### Diagnostic Criteria for Outcomes

Spontaneous preterm birth was defined as birth at either less than 37 weeks or less than 34 weeks with labor and/or spontaneous rupture of membranes. Iatrogenic preterm birth was defined as birth at either less than 37 weeks or less than 34 weeks without labor or spontaneous rupture of membranes. Preeclampsia and severe preeclampsia definitions aligned with American College of Obstetricians and Gynecologists criteria.^23^ As markers of hemolysis were not commonly used in clinical practice, HELLP syndrome was defined as a platelet count less than 100 × 10^3^/μL (to convert to 10^9^/L, multiply by 1) with aspartate transaminase (AST) and alanine transaminase (ALT), more than double the upper limit of the reference range for our laboratory (AST: ≥74 U/L; ALT: ≥80 U/L [to convert to microkatals per liter, multiply by 0.0167]). Birth weight percentiles were derived via Intergrowth-21st from gestational age and sex at birth.^24^ The diagnosis of gestational diabetes adhered to the Diabetes Canada Clinical Practice Guideline and included hemoglobin A_1c_ (HbA_1c_) greater than 5.8% (to convert to proportion of total hemoglobin, multiply by 0.01), which temporarily replaced glucose challenge testing at our institution during the COVID-19 pandemic.^25^ Given an anticipated final sample size of 9000, with an estimated 1% rate of preterm birth at less than 34 weeks and a 2% prevalence of low PlGF level (<100 pg/mL), we expected to have more than 90% power to detect a 5-fold difference in our primary outcome.^1,13^

### Statistical Analysis

Participant characteristics were compared using the χ^2^ or Fisher exact tests for categorical variables and the Wilcoxon rank sum test for continuous variables. Receiver operating characteristic (ROC) curves were constructed to determine the area under the curve (AUC) and optimal PlGF threshold, using the Youden Index, for early preterm birth. Test performance was assessed at our a priori threshold of 100 pg/mL,^21,22^ as well as various other PlGF levels, for sensitivity, specificity, positive predictive value (PPV), negative predictive value (NPV), positive likelihood ratio (LR), and negative LR. The Kaplan-Meier method was used to estimate survival curves, and the log-rank statistic was used to compare different PlGF thresholds and the primary end point of time-to-birth from PlGF screen. *P* values were 2-sided, and *P* < .05 was considered statistically significant.

The primary outcome, all early preterm birth at less than 34 weeks, was analyzed by log binomial regression using an a priori model, adjusting for known risk factors for placental dysfunction and preterm birth (maternal age, race, parity, prepregnancy weight, and gestational diabetes). The key exposure variable, PlGF level, was analyzed in several ways. For our main exposures of interest, PlGF was assessed as a binary variable with either PlGF level less than 100 pg/mL or below the ROC-derived threshold. We also examined PlGF as a continuous variable, quintiles, derived percentiles, and as a categorical variable.^13^ The secondary outcomes were analyzed by multinomial logistic regression, log binomial regression, or by linear regression, as appropriate.

The diagnosis of preeclampsia relied on inpatient EMRs from any pregnancy admission, including for birth. Two independent maternal-fetal medicine specialist coauthors (J.W.S. and J.C.K.) reviewed uncertain records and confirmed coding accuracy in a random selection of pregnancies.

Significant missingness over 10% was addressed by multiple imputation of 10 iterations. Unknown race was coded as its own category for multivariable modeling.

All analyses were performed in R version 4.3.3. Data were analyzed from January to May 2024.

## Results

### Participants

**Figure 1.**
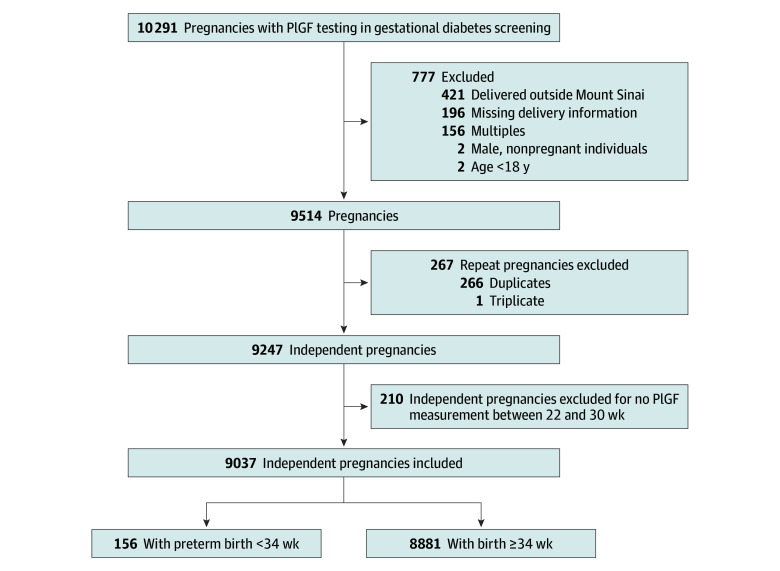
Study Recruitment Flowchart PlGF indicates placental growth factor.

Between April 2020 and April 2023, 10 291 pregnant individuals underwent a PlGF test with gestational diabetes screening. Of these, 7.6% were excluded following EMR review, the most common reason being birth outside Mount Sinai Hospital (Figure 1). The final sample included 9037 unique pregnant individuals.

### Baseline Characteristics

Participant characteristics are found in Table 1, stratified by PlGF level of 100 pg/mL, given the a priori clinical significance of this value.^13,21^ There were 8930 participants with a PlGF level of 100 pg/mL or greater and 107 participants with a PlGF level less than 100 pg/mL. Median (IQR) age at birth was 35 (32 to 37) years, and there were 627 Asian or South Asian individuals (7%), 221 Black individuals (2%), 1644 White individuals (18%), 1762 individuals who identified as other race (19%), and 4783 individuals with unknown race (53%). The high rate of unknown race reflected voluntary self-identification of race in the EMR. The low PlGF level cohort had significantly higher rates of advanced parity than the reference range PlGF level cohort (parity ≥4, 4% vs 1%; *P* = .02). The incidence of gestational diabetes was significantly higher in the low PlGF level group (14% vs 4%; *P* < .001), as was prepregnancy weight (median [IQR], 72.00 [61.10-86.80] vs 62.00 [56.00-71.00] kg; *P* < .001). Gestational age at PlGF test was approximately 4 days earlier in the low PlGF level vs reference range PlGF level cohort (median [IQR], 25.57 [24.50-27.21] weeks vs 26.14 [24.86-27.71]; *P* = .006) (eFigure 1 in Supplement 1); however, there was no significant difference in the proportion of tests between 24 and 28 weeks.

**Table 1.** Participant Characteristics Overall and by PlGF Level

Characteristic	Participants, No. (%)			*P* value^a^
	Overall (N = 9037)	PlGF level, pg/mL		
		≥100 (n = 8930)	<100 (n = 107)	
Maternal age at delivery, median (IQR), y	35.00 (32.00-37.00)	35.00 (31.00-38.00)	35.00 (31.00-38.00)	.87
Gravidity				
1	3689 (41)	3648 (41)	41 (38)	.07
2	2762 (31)	2738 (31)	24 (22)	
3	1449 (16)	1429 (16)	20 (19)	
4	653 (7)	641 (7)	12 (11)	
≥5	484 (5)	474 (5)	10 (9)	
Parity				
0	5141 (57)	5085 (57)	56 (52)	.02
1	2959 (33)	2926 (33)	33 (31)	
2	718 (8)	707 (8)	11 (10)	
3	151 (2)	148 (2)	3 (3)	
≥4	68 (1)	64 (1)	4 (4)	
Multiparity (parity >0)	3896 (43)	3845 (43)	51 (48)	.34
Maternal race				
Asian or South Asian	627 (7)	618 (7)	9 (8)	.12
Black	221 (2)	215 (2)	6 (6)	
White	1644 (18)	1630 (18)	14 (13)	
Other^b^	1762 (19)	1745 (20)	17 (16)	
Unknown	4783 (53)	4722 (53)	61 (57)	NA
Maternal height				
Median (IQR), m (n = 7174)	1.65 (1.60-1.70)	1.65 (1.60-1.70)	1.63 (1.57-1.67)	.09
Unknown	1863	1821	42	NA
Prepregnancy weight				<.001
Median (IQR), kg (n = 6087)	62.00 (56.00-71.00)	62.00 (56.00-71.00)	72.00 (61.10-86.80)	
Unknown	2950	2894	56	NA
Prepregnancy BMI, median (IQR)				
Median (IQR) (n = 5849)	22.95 (20.80-25.97)	22.92 (20.78-25.95)	27.06 (23.04-34.63)	<.001
Unknown	3188	3130	58	NA
Gestational diabetes				
Yes (n = 9032)	360(4.0)	345 (3.9)	15 (14)	<.001
Unknown	5	3	2	NA
PlGF, median (IQR), pg/mL	558.00 (375.00-820.00)	562.00 (382.00-825.00)	65.00 (38.00-85.00)	<.001
PlGF percentile by gestational age	65.79 (37.32-86.82)	66.29 (38.60-86.98)	0.50 (0.50-0.50)	<.001
Gestational age at PlGF test, median (IQR), wk	26.14 (24.86-27.71)	26.14 (24.86-27.71)	25.57 (24.50-27.21)	.006
Proportion of PlGF tests from 24 to 28 wk	7243 (80)	7152 (80)	91 (85)	.20

Abbreviations: BMI, body mass index (calculated as weight in kilograms divided by height in meters squared); NA, not applicable; PlGF, placental growth factor.

^a^ Wilcoxon rank sum test, Pearson χ^2^ test, or Fisher exact test.

^b^ Includes individuals who did not identify with any of the listed race categories.

Prepregnancy body mass index (BMI) was unavailable for 35% of participants. We performed multiple imputation on prepregnancy weight, rather than BMI, as it was more commonly available yet strongly associated with prepregnancy BMI. We adjusted for imputed prepregnancy weight in subsequent multivariable regression analyses.

### Primary Outcome: Early Preterm Birth 

**Figure 2.**
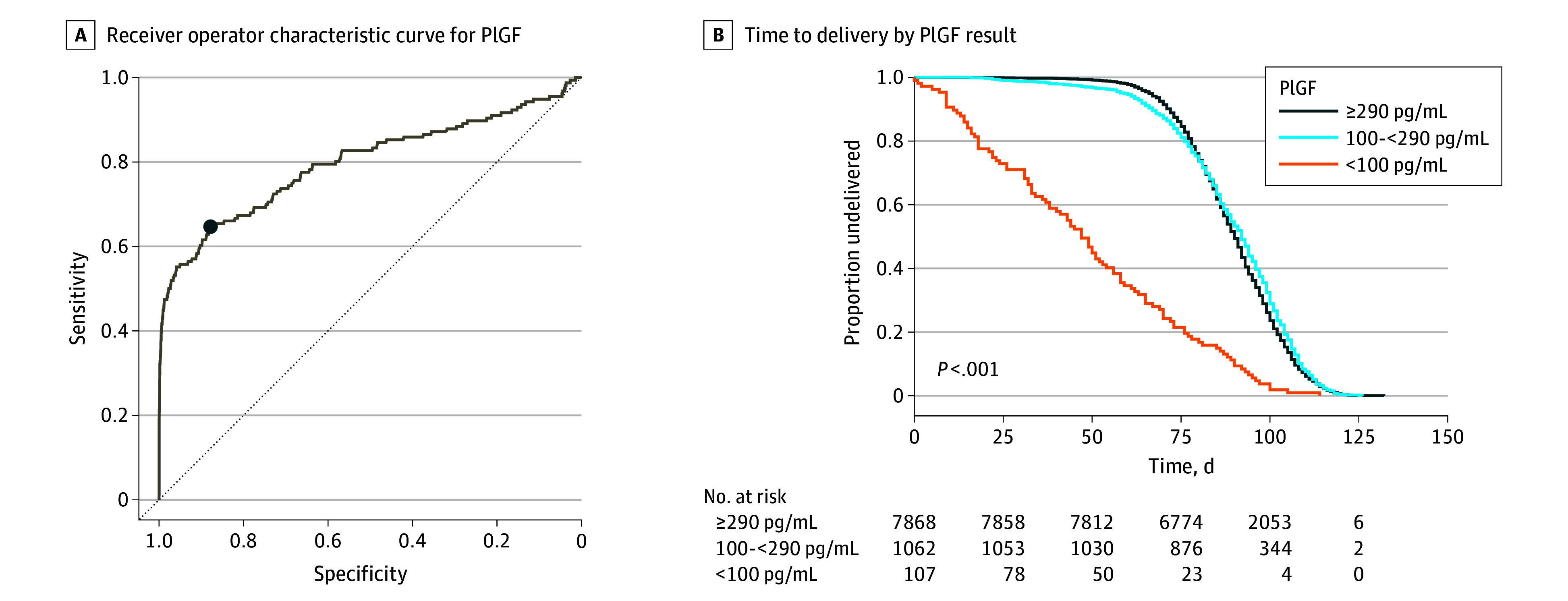
Screening Performance of Placental Growth Factor (PlGF) From 22 to 30 Weeks’ Gestation for Preterm Birth at Less Than 34 Weeks A, Receiver operator characteristic curve for PlGF in pg/mL, with an area under the curve of 0.80 (95% CI, 0.75-0.85). The optimal threshold by Youden Index (blue dot) was 290.5 pg/mL, with sensitivity 0.647 and specificity 0.879. B, Time to delivery in days from PlGF screen, stratified by PlGF test result less than 100, 100 to 289, and 290 or greater pg/mL.

In its raw value, PlGF demonstrated an AUC of 0.80 (95% CI, 0.75-0.85) (Figure 2A) for the primary outcome of early preterm birth at less than 34 weeks. The optimal PlGF threshold according to Youden Index was 290 pg/mL (decimals are not reported clinically), corresponding to a sensitivity of 64.7% (95% CI, 57.1%-72.4%), specificity of 87.9% (95% CI, 87.2%-88.5%), PPV of 8.6% (95% CI, 7.5%-9.6%), NPV of 99.3% (95% CI, 99.1%-99.4%), positive LR of 5.347 (95% CI, 4.339-6.537), and negative LR of 0.402 (95% CI, 0.314-0.531) for early preterm birth. Assigning a cutpoint of 290 pg/mL resulted in 12.9% of our cohort being categorized as having a low PlGF level. At our a priori threshold of 100 pg/mL, 1% of our cohort was categorized as having a low PlGF level, with a sensitivity of 39.7% (95% CI, 32.1%-47.4%), specificity of 99.5% (95% CI, 99.3%-99.6%), PPV of 57.9% (95% CI, 49.5%-67.0%), NPV of 98.9% (95% CI, 98.8%-99.1%), positive LR of 79.400 (95% CI, 53.434-115.137), and negative LR of 0.606 (95% CI, 0.494-0.742) for early preterm birth (eTable 1 in Supplement 1). PlGF expressed as gestational age–adjusted percentiles performed similarly to raw values: the AUC was 0.79 (95% CI, 0.74-0.84) (eFigure 2 in Supplement 1).

The Kaplan-Meier plot of 3 distinct groups (PlGF level <100, 100 to <290, and ≥290 pg/mL) revealed significantly lower delivery-free survival confined to the group with a PlGF level less than 100 pg/mL (Figure 2B). By 50 days from the PlGF test, more than 50% of the cohort with a PlGF level less than 100 pg/mL had given birth, compared with 3% in the 100 to less than 290 pg/mL cohort and less than 1% in the 290 pg/mL or greater cohort (Figure 2B).

At a PlGF threshold of less than 290 pg/mL, the adjusted relative risk for early preterm birth was 10.56 (95% CI, 7.57-14.73; *P* < .001). Among participants with a PlGF level less than 100 pg/mL, the risk of early preterm birth increased to 48.23 (95% CI, 36.55-63.64; *P* < .001). As the association between PlGF level and preterm birth was nonlinear, we converted PlGF levels to quintiles and found that the lowest quintile (10-343 pg/mL) was associated with a significantly increased risk of early preterm birth compared with the highest quintile (aRR, 6.18 [95% CI, 3.49-10.92]; *P* < .001). A dose-response trend was observed between PlGF percentile category and early preterm birth, with the relative risk increasing as PlGF percentile decreased, to a peak relative risk of 52.83 (95% CI, 36.60-76.25; *P* < .001) in the lower than 2.5th percentile group (Table 2). Even increases of 1 pg/mL or 1 gestational age–adjusted percentile resulted in statistically significant decreases in the risk of early preterm birth (eTable 2 in Supplement 1).

**Table 2.** Unadjusted and Adjusted Relative Risks for Early Preterm Birth at Less Than 34 Weeks by PlGF Model Inputs

PlGF model input	Participants, No.	Univariable		Multivariable	
		Unadjusted relative risk (95% CI)	*P* value	Adjusted relative risk (95% CI)	*P* value
PlGF quintiles, decreasing (pg/mL)					
5 (903 to 4931)	1813	1 [Reference]	NA	1 [Reference]	NA
4 (641 to 902)	1806	0.57 (0.24-1.36)	.20	0.54 (0.23-1.29)^a^	.17
3 (482 to 640)	1802	0.72 (0.32-1.61)	.42	0.66 (0.29-1.49)^a^	.32
2 (344 to 481)	1809	1.36 (0.68-2.70)	.38	1.21 (0.61-2.43)^a^	.58
1 (10 to 343)	1806	7.47 (4.29-12.99)	<.001	6.18 (3.49-10.92)^a^	<.001
Dichotomous <290 pg/mL	1169	12.02 (8.72-16.57)	<.001	1.56 (7.57-14.73)^a^	<.001
Dichotomous <100 pg/mL	107	55.05 (42.53-71.24)	<.001	48.23 (36.55-63.64)	<.001
Categorical (percentile)^13^					
≥50	5987	1 [Reference]	NA	1 [Reference]	NA
10 to <50	2533	1.85 (1.17-2.92)	.009	1.85 (1.17-2.93)^b^	.009
5 to <10	217	2.69 (0.97-7.45)	.06	2.76 (1.00-7.65)^b^	.05
2.5 to <5	118	9.90 (4.75-20.65)	<.001	9.33 (4.45-19.60)^b^	<.001
<2.5	182	56.97 (39.94-81.25)	<.001	52.83 (36.60-76.25)^b^	<.001
Dichotomous <2.5th percentile^13^	182	40.64 (30.75-53.71)	<.001	37.33 (27.85-5.03)^b^	<.001

Abbreviations: NA, not applicable; PlGF, placental growth factor.

^a^ Adjusted for maternal age, race, parity, prepregnancy weight, gestational diabetes, and gestational age of PlGF test.

^b^ Adjusted for maternal age, race, parity, prepregnancy weight, and gestational diabetes.

### Secondary Outcomes

Secondary outcomes, stratified by PlGF level less than 100 pg/mL, are reported in Table 3. The adjusted relative risks for secondary outcomes were significantly higher in the low PlGF level cohort for all but spontaneous preterm birth at less than 37 weeks. Among the low PlGF level cohort, the adjusted relative risk for iatrogenic early preterm birth was 92.11 (95% CI, 64.83-130.87; *P* < .001) and the adjusted difference in mean gestational age at birth was 6.10 (95% CI, 5.80-6.39) weeks earlier compared with the reference range PlGF level cohort (*P* < .001), while the rate of stillbirth was more than 10% (aRR, 36.78 [95% CI, 18.63-72.60]; *P* < .001), and the rate of preeclampsia, nearly 50% (aRR, 11.01 [95% CI, 8.85-13.69]; *P* < .001). Frequency of Cesarean birth was significantly higher among the low PlGF level cohort than the reference range PlGF level cohort (aRR, 4.04 [95% CI, 2.60-6.29]; *P* < .001). ROC curves for secondary outcomes are presented in eFigure 3 in Supplement 1. Notably, the AUC for iatrogenic early preterm birth was 0.90 (95% CI, 0.85-0.94) (eFigure 3 in Supplement 1).

**Table 3.** Secondary Outcomes Overall and by PlGF Level

Outcome	Events, No. (%)			Adjusted relative risk (95% CI)	*P* value
	Overall (N = 9037)	PlGF, mg/mL			
		≥100 (n = 8930)	<100 (n = 107)		
Gestational age at birth, median (IQR), wk	39.14 (37.43-40.86)	39.14 (37.43-40.86)	32.71 (25.21-40.21)	−6.10 (−6.39 to −5.80)^a^^,^^b^	<.001
Preterm birth <37 wk	622 (6.9)	539 (6.0)	83 (77.6)	12.89 (11.32 to 14.68)^c^	<.001
Spontaneous preterm birth <37 wk	329 (3.6)	323 (3.6)	6 (5.6)	1.19 (0.52 to 2.73)^a^	.68
Spontaneous preterm birth <34 wk	52 (0.6)	47 (0.5)	5 (4.7)	5.98 (2.33 to 15.37)^a^	<.001
Iatrogenic preterm birth <37 wk	293 (3.2)	216 (2.5)	77 (72.0)	29.45 (24.65 to 35.20)^c^	<.001
Iatrogenic preterm birth <34 wk	104 (1.2)	47 (0.5)	57 (53.3)	92.11 (64.83 to 130.87)^a^	<.001
Stillbirth	36 (0.4)	25 (0.3)	11 (10.3)	36.78 (18.63-72.60)^c^	<.001
Preeclampsia	437 (4.8)	384 (4.3)	53 (49.5)	11.01 (8.85 to 13.69)^c^	<.001
Severe preeclampsia	142 (1.6)	120 (1.2)	22 (20.6)	10.46 (6.51 to 16.82)^a^	<.001
HELLP syndrome	8 (0.09)	7 (0.08)	1 (0.93)	NA^d^	NA^d^
SGA, percentile					
<10th	595 (6.6)	542 (6.1)	53 (50.0)	8.00 (6.49 to 9.86)^c^	<.001
<3rd	169 (1.9)	133 (1.5)	36 (34.0)	22.54 (16.25 to 31.27)^a^	<.001
Mode of birth					
Spontaneous vaginal	4822 (53.4)	4793 (53.7)	29 (27.1)	1 [Reference]	NA
Assisted vaginal	1031 (11.4)	1027 (11.5)	4 (3.7)	0.74 (.26 to 2.13)^a^	.57
Cesarean	3184 (35.2)	3110 (34.8)	74 (69.2)	4.04 (2.60 to 6.29)^a^	<.001

Abbreviations: NA, not applicable; HELLP, hemolysis, elevated liver-enzyme levels, and low platelet count; PlGF, placental growth factor; SGA, small for gestational age.

^a^ Adjusted for: maternal age, race, parity, prepregnancy weight, gestational diabetes, gestational age of PlGF test.

^b^ β Coefficient from multivariable linear regression.

^c^ Adjusted for maternal age. Given low event rates, adjustment for all covariates was not possible. To address this, we ran bivariable models with each of the following covariates, in addition to PlGF level less than 100 pg/mL: age, race, parity, prepregnancy weight, gestational diabetes, and gestational age of PlGF test. There was no notable confounding in any model. Thus, only maternal age-adjusted relative risks are included here.

^d^ There were too few HELLP syndrome events for modeling.

### Sensitivity Analyses

Our primary and secondary outcome findings remained consistent across nulliparous participants (eTable 3 in Supplement 1), participants with known maternal race (eTable 4 in Supplement 1), and participants with prepregnancy BMI recorded (eTable 5 in Supplement 1). Our results were also consistent in multivariable regression without adjustment for weight or BMI (eTable 6 in Supplement 1).

## Discussion

In this large, prospective cohort study, low PlGF level at the time of gestational diabetes screening was strongly associated with all early preterm birth at less than 34 weeks’ gestation in unselected, singleton pregnancies. In exploratory secondary analysis, PlGF screening had the best discrimination for iatrogenic early preterm birth at less than 34 weeks, with an AUC of 0.90. Importantly, raw PlGF test performance in picograms per milliliter was indistinguishable from gestational age–adjusted PlGF percentiles, and the association with early preterm birth was unaffected by maternal age, parity, or subgroup analysis of participants who self-reported race. Our observed rates of adverse outcomes align with those reported in the general population, thereby strengthening the generalizability of our single-center results and validating the unselected nature of our study population. These findings demonstrate that PlGF testing in the middle trimester is a promising unimodal strategy to screen for risk of early preterm birth in the general obstetrical population. Future investigation, including via randomized clinical trial design and cost-effectiveness analysis, is warranted.

Although by Youden Index, 290 pg/mL was the optimal PlGF threshold for early preterm birth, we conclude that our a priori threshold of 100 pg/mL is the most clinically relevant universal screening cutoff for several reasons. First, a PlGF level less than 100 pg/mL conferred a positive LR of 79.40 for early preterm birth, maintaining an NPV of 99%. Second, participants with a PlGF level less than 100 pg/mL gave birth significantly earlier than those with a PlGF level of 100 to less than 290 pg/mL or 290 pg/mL or greater, indicating that the risk of early preterm birth observed with a PlGF level less than 290 pg/mL was largely driven by individuals with a PlGF level less than 100 pg/mL. Finally, at a threshold of 100 pg/mL, low PlGF level was associated with dramatically increased risks for stillbirth, severe preeclampsia, and SGA birthweight. Screening for PlGF level less than 100 pg/mL identified most individuals who would go on to experience iatrogenic early preterm birth and nearly one-third of all individuals who had stillbirths. Collectively, these maternal and fetal risks were associated with a 6-week lower mean gestational age at birth and a 4-fold increase in the need for Cesarean birth, reflecting the strong association with iatrogenic preterm birth and likely underlying placental disease.

This single blood test, timed with an existing universal screening program for gestational diabetes, has the potential to revolutionize routine prenatal care. Weekly or alternating week clinic visits, designed decades ago to detect either hypertension or suboptimal fetal growth, are imprecise, time-consuming, and, therefore, costly to deliver.^26^ The addition of PlGF testing to current gestational diabetes screening could rule out clinically significant early preterm birth with 99% NPV. We envision a streamlined, enhanced care pathway for pregnant people with a PlGF level less than 100 pg/mL, to mitigate against the worst consequences of early preterm birth and its associated conditions.^9^ For example, timely access to antihypertensive therapies and effective maternal education, including home blood pressure monitoring, could substantially reduce the high incidence of complications arising from hypertensive diseases of pregnancy.^27,28^ Enhanced maternal-fetal surveillance among individuals with positive screening results could prevent the unacceptably high rate of stillbirth prior to labor afflicting all health care systems, including those with liberal access to fetal ultrasonographic examinations.^29^ By extension, PlGF screening could empower patients with values of 100 pg/mL or greater, especially those without ready access to tertiary obstetrical centers, to receive care closer to home. Strategically redirecting resources to individuals whose screening results suggest they are at highest risk for early preterm birth could improve families’ experiences of pregnancy, prevent morbidity and mortality, and save substantially on overall health care costs.

### Limitations

This study has some limitations. A significant limitation of our study is lack of blinding. The dramatic onset of the COVID-19 pandemic incentivized pregnant patients and clinicians to minimize in-person visits.^30^ With compelling evidence of the association between PlGF level less than 100 pg/mL and the development of preeclampsia,^21,31,32^ we received REB approval for opt-out PlGF testing with gestational diabetes screening, encouraging patients with reference range values to receive ongoing virtual care until 36 weeks. We speculate that the true rate of stillbirth associated with PlGF level less than 100 pg/mL could be higher than reported here, as most patients with positive screening results received specialized care designed to prevent antepartum stillbirth. Conversely, clinician awareness of a patient’s low PlGF level could have influenced the timing of iatrogenic birth toward an earlier gestational age, thereby exaggerating our findings. Nonetheless, given that the rate of early preterm birth differed so dramatically at the PlGF threshold of less than 100 pg/mL, we doubt the study design unduly influenced our findings. Furthermore, the rate of spontaneous early preterm birth, which is not subject to clinician discretion, was also increased among participants with PlGF level less than 100 pg/mL, as previously reported.^33^

Another limitation of our study, which was conducted in a diverse, urban population, was that only half of participants self-reported race within our EMR. While subgroup analysis showed no association of race category with test performance, we remain cautious in our data interpretation pending similar studies being published in other health care settings. As an example, in a recent large-scale UK study of PlGF screening at 35 to 36 weeks’ gestation, designed to estimate the development of preeclampsia at term, median PlGF levels were significantly higher in Black individuals compared with East Asian, South Asian, and White individuals as well as those who identified as multiple races. Should the trajectory of PlGF levels remain higher across earlier gestational ages in Black pregnant people, this observation may disadvantage Black individuals with higher false-negative test rates, unless their PlGF test results are specifically adjusted for this race category.^34^

## Conclusions

Our large-scale, prospective cohort study of midpregnancy PlGF screening for early preterm birth at less than 34 weeks achieved high discriminatory performance, especially for iatrogenic births, which make up most early preterm births. A screening PlGF level less than 100 pg/mL was present in 40% of all early preterm births, more than 50% of iatrogenic early preterm births, and more than 30% of stillbirths. The test is robust across a 24- to 28-week window, aligned with gestational diabetes screening, and without need for gestational age adjustment or consideration of maternal characteristics. As such, this test could be widely introduced and easily understood by clinicians, who in turn can focus on delivering evidence-based interventions to mitigate against adverse outcomes associated with early preterm birth. Future randomized trials are needed to determine the impact of implementing universal PlGF screening.

## Notes

Supplement 1. **eFigure 1.** Frequency Plot of Gestational Age at PlGF Test **eFigure 2.** Receiver Operator Characteristic (ROC) Curve for All PTB<34 Weeks by PlGF as Gestational Age-Adjusted Percentile **eFigure 3.** Receiver Operator Characteristic (ROC) Curves for Secondary Outcomes **eTable 1.** Screening Performance of PlGF at ROC-Derived and A Priori Thresholds **eTable 2.** Unadjusted and Adjusted Relative Risks for Preterm Birth <34 Weeks by Continuous PlGF (pg/mL and Gestational Age-Adjusted Percentiles) **eTable 3.** Summary of Sensitivity Analysis of Primary Outcome, Preterm Birth <34 Weeks, Among Nulliparous (Parity = 0) Participants (n = 5141) **eTable 4.** Summary of Sensitivity Analysis of Primary Outcome, Preterm Birth <34 Weeks, Among Participants With Maternal Race Known (n = 4254) **eTable 5.** Summary of Sensitivity Analysis of Primary Outcome, Preterm Birth <34 Weeks, Among Participants With Prepregnancy BMI Known (n = 5849) **eTable 6.** Summary of Sensitivity Analysis of Primary Outcome, Preterm Birth <34 Weeks, Without Adjustment for Prepregnancy BMI or Prepregnancy Weight (n = 9037)

Supplement 2.
Data Sharing Statement
