# Supplementary material for: Midpregnancy Placental Growth Factor Screening and Early Preterm Birth
Source: JAMA Netw Open. 2024 Nov 14;7(11):e2444454. doi: 10.1001/jamanetworkopen.2024.44454 (PMC11565265; doi:10.1001/jamanetworkopen.2024.44454)
Supplement: Supplement 1. — eFigure 1. Frequency Plot of Gestational Age at PlGF Test eFigure 2. Receiver Operator Characteristic (ROC) Curve for All PTB<34 Weeks by PlGF as Gestational Age-Adjusted Percentile eFigure 3. Receiver Operator Characteristic (ROC) Curves for Secondary Outcomes eTable 1. Screening Performance of PlGF at ROC-Derived and A Priori Thresholds eTable 2. Unadjusted and Adjusted Relative Risks for Preterm Birth <34 Weeks by Continuous PlGF (pg/mL and Gestational Age-Adjusted Percentiles) eTable 3. Summary of Sensitivity Analysis of Primary Outcome, Preterm Birth <34 Weeks, Among Nulliparous (Parity = 0) Participants (n = 5141) eTable 4. Summary of Sensitivity Analysis of Primary Outcome, Preterm Birth <34 Weeks, Among Participants With Maternal Race Known (n = 4254) eTable 5. Summary of Sensitivity Analysis of Primary Outcome, Preterm Birth <34 Weeks, Among Participants With Prepregnancy BMI Known (n = 5849) eTable 6. Summary of Sensitivity Analysis of Primary Outcome, Preterm Birth <34 Weeks, Without Adjustment for Prepregnancy BMI or Prepregnancy Weight (n = 9037) [file jamanetwopen-e2444454-s001.pdf]

Supplemental Online Content

Gladstone RA, Ahmed S, Huszti E, et al. Midpregnancy placental growth factor screening and early preterm birth. *JAMA Netw Open*. 2024;7(11):e2444454. doi:10.1001/jamanetworkopen.2024.44454

eFigure 1. Frequency Plot of Gestational Age at PlGF Test
eFigure 2. Receiver Operator Characteristic (ROC) Curve for All PTB<34 Weeks by PlGF as Gestational Age-Adjusted Percentile
eFigure 3. Receiver Operator Characteristic (ROC) Curves for Secondary Outcomes
eTable 1. Screening Performance of PlGF at ROC-Derived and A Priori Thresholds
eTable 2. Unadjusted and Adjusted Relative Risks for Preterm Birth <34 Weeks by Continuous PlGF (pg/mL and Gestational Age-Adjusted Percentiles)
eTable 3. Summary of Sensitivity Analysis of Primary Outcome, Preterm Birth <34 Weeks, Among Nulliparous (Parity = 0) Participants (n = 5141)
eTable 4. Summary of Sensitivity Analysis of Primary Outcome, Preterm Birth <34 Weeks, Among Participants With Maternal Race Known (n = 4254)
eTable 5. Summary of Sensitivity Analysis of Primary Outcome, Preterm Birth <34 Weeks, Among Participants With Prepregnancy BMI Known (n = 5849)
eTable 6. Summary of Sensitivity Analysis of Primary Outcome, Preterm Birth <34 Weeks, Without Adjustment for Prepregnancy BMI or Prepregnancy Weight (n = 9037)

This supplemental material has been provided by the authors to give readers additional information about their work.

© 2024 Gladstone RA et al. *JAMA Network Open*.

eFigure 1: Frequency plot of gestational age at PlGF test

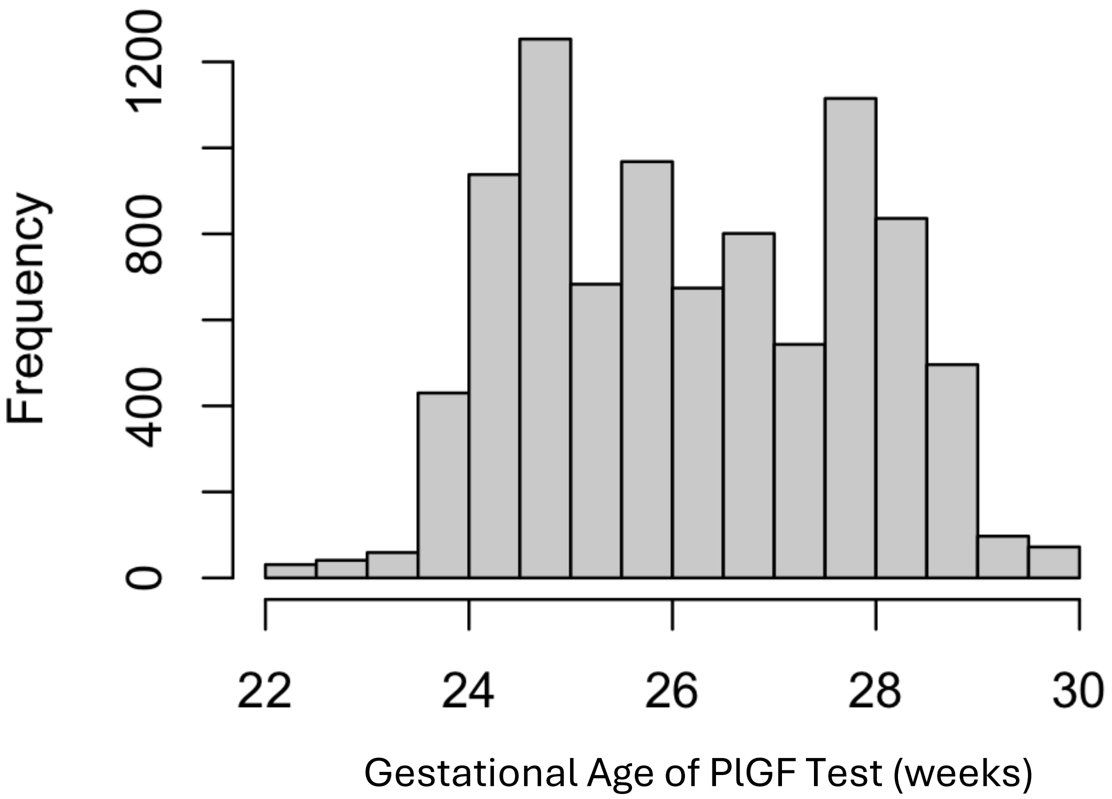

© 2024 Gladstone RA et al. *JAMA Network Open*.

eFigure 2: Receiver operator characteristic (ROC) curve for all PTB <34 weeks by PlGF as gestational age-adjusted percentile[a]

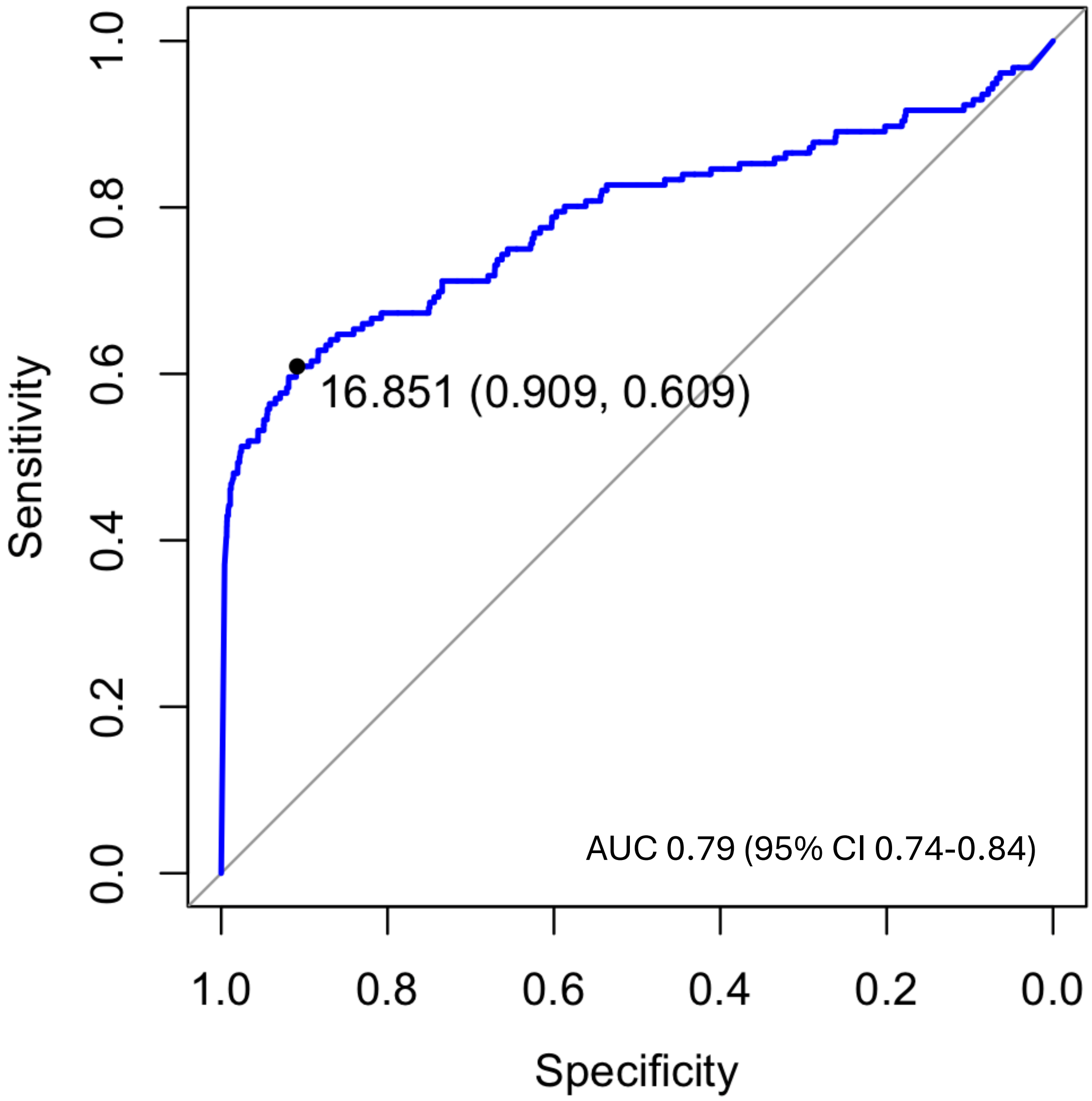

[a] Optimal threshold by Youden's Index noted, with specificity and sensitivity in parentheses.

© 2024 Gladstone RA et al. *JAMA Network Open*.

eFigure 3: Receiver operator characteristic (ROC) curves for secondary outcomes[a]

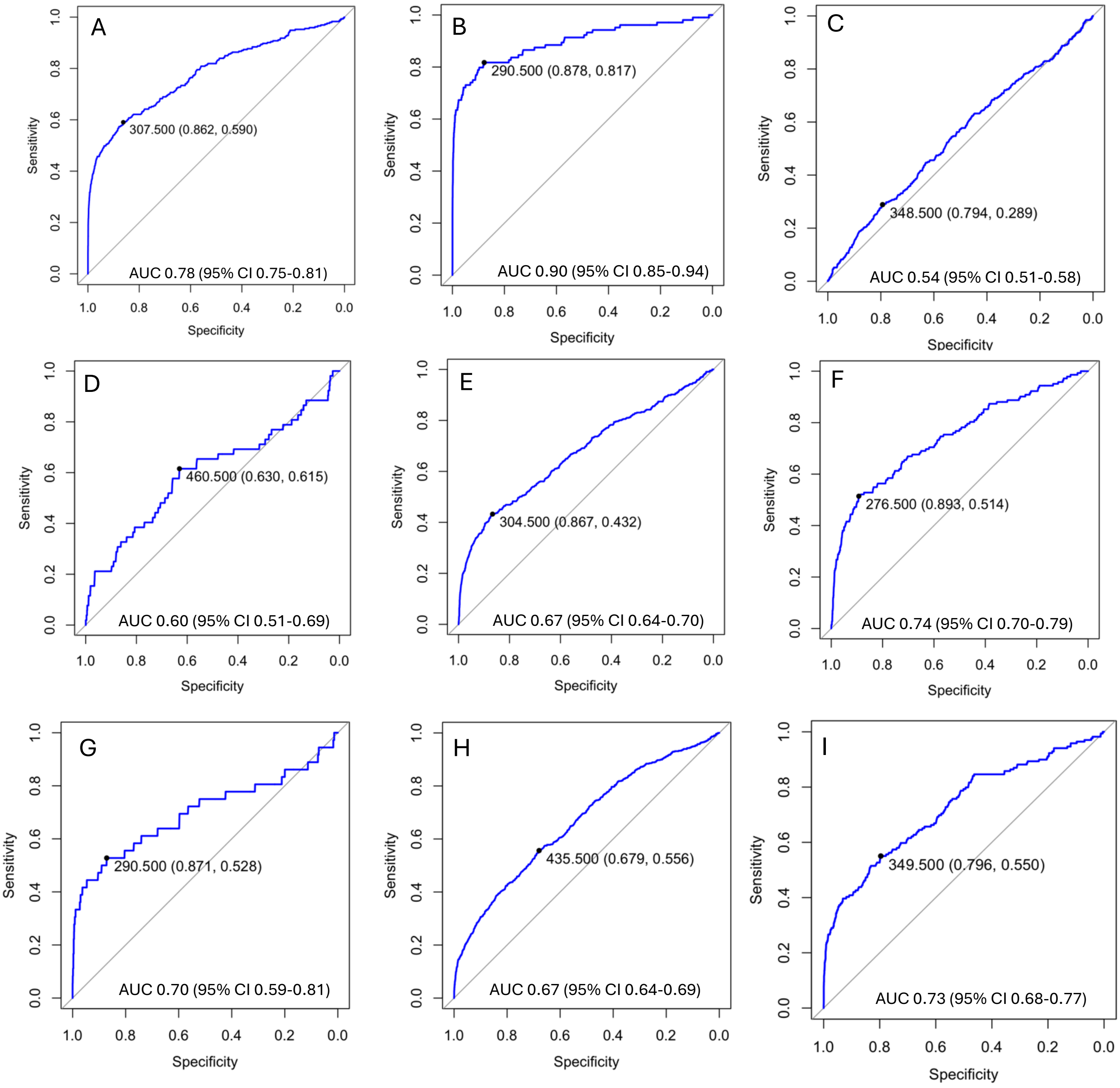

[a] Optimal threshold by Youden's Index noted, with specificity and sensitivity in parentheses. (A) Iatrogenic preterm birth <37 weeks (n=622), (B) iatrogenic preterm birth <34 weeks (n=104), (C) spontaneous preterm birth <37 weeks (n=329), (D) spontaneous preterm birth <34 weeks (n=52), (E) preeclampsia (n=437), (F) severe preeclampsia (n=142), (G) stillbirth (n=36), (H) small for gestational age <10th percentile (n=595), (I) small for gestational age birth <3rd percentile (n=169).

© 2024 Gladstone RA et al. *JAMA Network Open.*

eTable 1: Screening performance of PlGF at ROC-derived and a priori thresholds

PlGF (pg/mL)	Screen Positive n (%)	Sensitivity	Specificity	PPV	NPV	LR+	LR-
290	1169 (12.9%)	0.647	0.879	0.086	0.993	5.347	0.402
100	107 (1.2%)	0.397	0.995	0.579	0.989	79.400	0.606

The screen positive rate, sensitivity, specificity, positive predictive value (PPV), negative predictive value (NPV), positive likelihood ratio (LR+), and negative likelihood ratio (LR-) for 290 pg/mL (the derived optimal threshold from the ROC curve) and 100 pg/mL (an established optimal diagnostic threshold value for suspected preeclampsia).

© 2024 Gladstone RA et al. *JAMA Network Open*.

eTable 2: Unadjusted and adjusted relative risks for preterm birth <34 weeks by continuous PlGF (pg/mL and gestational age-adjusted percentiles)

**PlGF Model Input**	**Univariable**		**Multivariable**	
	Unadjusted Relative Risk (95% CI)	P value	Adjusted Relative Risk (95% CI)	P value
Continuous PlGF (pg/mL) (N=9,037)	0.994 (0.993-0.995)	<0.001	0.995 (0.994-0.996)[a]	<0.001
Continuous gestational age-adjusted percentiles (N=9,037)	0.957 (0.951-0.963)	<0.001	0.958 (0.952-0.965)[b]	<0.001

[a]Adjusted for: maternal age, race, parity, pre-pregnancy weight, gestational diabetes, gestational age of PlGF test

[b]Adjusted for: maternal age, race, parity, pre-pregnancy weight, gestational diabetes

CI: Confidence Interval

© 2024 Gladstone RA et al. *JAMA Network Open*.

eTable 3: Summary of sensitivity analysis of primary outcome, preterm birth <34 weeks, among nulliparous (parity = 0) participants (n=5,141)

**PlGF Model Input**		**Univariable**		**Multivariable**	
		Unadjusted Relative Risk (95% CI)	P value	Adjusted Relative Risk[a] (95% CI)	P value
Quintiles, decreasing (pg/mL)	5 (903 to 4931)	Reference	Reference	Reference	Reference
	4 (641 to 902)	0.75 (0.24-2.35)	0.62	0.75 (0.24-2.36)	0.62
	3 (482 to 640)	0.73 (0.23-2.29)	0.59	0.74 (0.23-2.31)	0.60
	2 (344 to 481)	1.14 (0.41-3.13)	0.80	1.14 (0.42-3.14)	0.79
	1 (17 to 343)	7.30 (3.33-16.01)	<0.001	7.34 (3.35-16.09)	<0.001
Dichotomous < 290 pg/mL		11.08 (7.04-17.45)	<0.001	11.07 (7.03-17.42)	<0.001
Dichotomous < 100 pg/mL		54.86 (37.58-80.09)	<0.001	54.76 (37.52-79.93)	<0.001
Categorical (percentile)[18]	≥ 50	Reference	Reference	Reference	Reference
	10 to <50	1.49 (0.74-2.99)	0.26	1.49 (0.74-2.99)	0.26
	5 to <10	3.92 (1.18-13.04)	0.03	3.91 (1.18-12.98)	0.03
	2·5 to <5	13.39 (5.199-34.55)	<0.001	13.33 (5.17-34.37)	<0.001
	<2·5	59.56 (35.77-99.17)	<0.001	59.55 (35.77-99.14)	<0.001
Dichotomous < 2·5th percentile[18]		43.38 (29.01-64.87)	<0.001	43.37 (29.01-64.84)	<0.001

[a]Adjusted for: maternal age. Given low event rates, adjustment for all covariates was not possible. To address this, we ran bivariable models with each of the following covariates, in addition to PlGF as relevant: age, race, parity, pre-pregnancy weight, gestational diabetes, and gestational age of PlGF test. There was no notable confounding in any model. Thus, only maternal age-adjusted relative risks are included here. CI: Confidence Interval

© 2024 Gladstone RA et al. *JAMA Network Open*.

eTable 4: Summary of sensitivity analysis of primary outcome, preterm birth <34 weeks, among participants with maternal race known (n=4,254)

**PlGF Model Input**		**Univariable**		**Multivariable**	
		Unadjusted Relative Risk (95% CI)	P value	Adjusted Relative Risk (95% CI)	P value
Quintiles, decreasing (pg/mL)	5 (903 to 4605)	Reference	Reference	Reference	Reference
	4 (641 to 902)	0.57 (0.24-1.36)	0.20	0.48 (0.12-1.91)[a]	0.30
	3 (482 to 640)	0.72 (0.32-1.61)	0.42	0.65 (0.18-2.29)[a]	0.50
	2 (344 to 481)	1.36 (0.68-2.70)	0.38	1.33 (0.47-3.76)[a]	0.59
	1 (11 to 343)	7.40 (4.25-12.88)	<0.001	6.88 (2.89-16.41)[a]	<0.001
Dichotomous < 290 pg/mL		12.02 (8.72-16.57)	<0.001	10.32 (6.26-17.01)[a]	<0.001
Dichotomous < 100 pg/mL		55.31 (37.42-81.75)	<0.001	47.11 (30.82-72.01)[a]	<0.001
Categorical (percentile)[18]	≥ 50	Reference	Reference	Reference	Reference
	10 to <50	2.38 (1.22-4.65)	0.01	2.41 (1.23-4.71)[b]	0.01
	5 to <10	4.80 (1.43-16.12)	0.01	5.12 (1.51-17.34)[b]	0.009
	2·5 to <5	8.60 (2.59-28.55)	<0.001	8.35 (2.49-27.99)[b]	<0.001
	<2·5	62.65 (35.99-109.05)	<0.001	58.36 (33.01-103.19)[b]	<0.001
Dichotomous < 2·5th percentile[18]		39.33 (25.80-59.95)	<0.001	36.54 (23.55-56.72)[b]	<0.001

[a]Adjusted for: maternal age, race, parity, pre-pregnancy weight, gestational diabetes, gestational age of PlGF test
[b]Adjusted for: maternal age, race, parity, pre-pregnancy weight, gestational diabetes
CI: Confidence Interval

© 2024 Gladstone RA et al. *JAMA Network Open*.

eTable 5: Summary of sensitivity analysis of primary outcome, preterm birth <34 weeks, among participants with pre-pregnancy BMI known (n=5,849)

**PlGF Model Input**		**Univariable**		**Multivariable**	
		Unadjusted Relative Risk (95% CI)	P value	Adjusted Relative Risk (95% CI)	P value
Quintiles, decreasing (pg/mL)	5 (904 to 4931)	Reference	Reference	Reference	Reference
	4 (641 to 902)	0.37 (0.10-1.39)	0.14	0.35 (0.09-1.33)[a]	0.12
	3 (482 to 640)	0.37 (0.10-1.40)	0.14	0.35 (0.09-1.30)[a]	0.12
	2 (344 to 481)	0.50 (0.15-1.65)	0.25	0.44 (0.13-1.46)[a]	0.18
	1 (10 to 343)	5.77 (2.73-12.19)	<0.001	4.26 (1.94-9.35)[a]	<0.001
Dichotomous < 290 pg/mL		14.11 (8.41-23.69)	<0.001	11.26 (6.51-19.49)[a]	<0.001
Dichotomous < 100 pg/mL		72.84 (47.69-111.27)	<0.001	60.09 (37.20-97.05)[a]	<0.001
Categorical (percentile)[18]	≥ 50	Reference	Reference	Reference	Reference
	10 to <50	1.75 (0.83-3.69)	0.14	1.71 (0.81-3.61)[b]	0.16
	5 to <10	3.62 (0.84-15.60)	0.08	3.60 (0.84-15.52)[b]	0.09
	2·5 to <5	17.59 (6.63-46.65)	<0.001	14.98 (5.57-40.32)[b]	<0.001
	<2·5	69.34 (38.80-123.92)	<0.001	55.57 (29.80-103.64)[b]	<0.001
Dichotomous < 2·5th percentile[18]		46.95 (29.78-74.00)	<0.001	36.68 (22.09-60.90)[b]	<0.001

[a]Adjusted for: maternal age, race, parity, gestational diabetes, gestational age of PlGF test
[b]Adjusted for: maternal age, race, parity, gestational diabetes
CI: Confidence Interval

© 2024 Gladstone RA et al. *JAMA Network Open.*

eTable 6: Summary of sensitivity analysis of primary outcome, preterm birth <34 weeks, without adjustment for pre-pregnancy BMI or pre-pregnancy weight (n=9,037)

**PlGF Model Input**		**Univariable**		**Multivariable**	
		Unadjusted Relative Risk (95% CI)	P value	Adjusted Relative Risk (95% CI)	P value
Quintiles, decreasing (pg/mL)	5 (903 to 4931)	Reference	Reference	Reference	Reference
	4 (641 to 902)	0.57 (0.24-1.36)	0.20	0.54 (0.23-1.30)[a]	0.17
	3 (482 to 640)	0.72 (0.32-1.61)	0.42	0.67 (0.30-1.50)[a]	0.33
	2 (344 to 481)	1.36 (0.68-2.70)	0.38	1.16 (0.58-2.35)[a]	0.67
	1 (10 to 343)	7.40 (4.25-12.88)	<0.001	6.15 (3.49-10.85)[a]	<0.001
Dichotomous < 290 pg/mL		12.02 (8.72-16.57)	<0.001	10.63 (7.62-14.82)[a]	<0.001
Dichotomous < 100 pg/mL		55.05 (42.53-71.24)	<0.001	48.06 (36.30-63.63)[a]	<0.001
Categorical (percentile)[18]	≥ 50	Reference	Reference	Reference	Reference
	10 to <50	1.85 (1.17-2.92)	0.009	1.89 (1.19-3.00)[b]	0.007
	5 to <10	2.69 (0.97-7.45)	0.06	2.82 (1.02-7.80)[b]	0.046
	2·5 to <5	9.90 (4.75-20.65)	<0.001	9.61 (4.59-20.11)[b]	<0.001
	<2·5	56.97 (39.94-81.25)	<0.001	52.82 (36.55-76.33)[b]	<0.001
Dichotomous < 2·5th percentile[18]		40.64 (30.75-53.71)	<0.001	37.00 (27.60-49.61)[b]	<0.001

[a]Adjusted for: maternal age, race, parity, gestational diabetes, gestational age of PlGF test
[b]Adjusted for: maternal age, race, parity, gestational diabetes
CI: Confidence Interval

© 2024 Gladstone RA et al. *JAMA Network Open*.
