# Supplementary material for: Midpregnancy Placental Growth Factor Screening and Early Preterm Birth
Source: JAMA Netw Open. 2024 Nov 14;7(11):e2444454. doi: 10.1001/jamanetworkopen.2024.44454 (PMC11565265; doi:10.1001/jamanetworkopen.2024.44454)
Supplement: Supplement 2. — Data Sharing Statement [file jamanetwopen-e2444454-s002.pdf]

Data Sharing Statement

Gladstone. Midpregnancy Placental Growth Factor Screening and Early Preterm Birth. *JAMA Netw Open*. Published November 12, 2024. doi:10.1001/jamanetworkopen.2024.44454

Data
Data available: Yes
Data types: Deidentified participant data, Data dictionary, Other (please specify)
Additional Information: Study protocol
How to access data: Deidentified participant data, data dictionary, and study protocol will be made available following review of requests made in writing to the Senior Author, Dr. John Kingdom.
When available: With publication

Supporting Documents
Document types: Statistical/analytic code
How to access documents: Statistical code will be made available following review of requests made in writing to the Senior Author, Dr. John Kingdom.
When available: With publication

Additional Information
Who can access the data: The data will be made available to researchers whose proposed use of the data has been approved.
Types of analyses: For any approved research.
Mechanisms of data availability: Data will be made available after approval of a proposal and with a signed data access agreement.
